# Carbon assimilation and distribution in cotton photosynthetic organs is a limiting factor affecting boll weight formation under drought

**DOI:** 10.3389/fpls.2022.1001940

**Published:** 2022-09-21

**Authors:** Jie Zou, Wei Hu, Dimitra A. Loka, John L. Snider, Honghai Zhu, Yuxia Li, Jiaqi He, Youhua Wang, Zhiguo Zhou

**Affiliations:** ^1^ Key Laboratory of Crop Growth Regulation, Ministry of Agriculture, Nanjing Agricultural University, Nanjing, China; ^2^ Institute of Industrial and Forage Crops, Hellenic Agricultural Organization, Larissa, Greece; ^3^ Department of Crop and Soil Sciences, University of Georgia, Tifton, GA, United States

**Keywords:** cotton, drought, photosynthetic organ, carbon assimilation, distribution, boll weight

## Abstract

Previous studies have documented cotton boll weight reductions under drought, but the relative importance of the subtending leaf, bracts and capsule wall in driving drought-induced reductions in boll mass has received limited attention. To investigate the role of carbon metabolism in driving organ-specific differences in contribution to boll weight formation, under drought conditions. Controlled experiments were carried out under soil relative water content (SRWC) (75 ± 5)% (well-watered conditions, control), (60 ± 5)% (moderate drought) and (45 ± 5)% (severe drought) in 2018 and 2019 with two cultivars Yuzaomian 9110 and Dexiamian 1. Under severe drought, the decreases of photosynthetic rate (*Pn*) and carbon isotope composition (δ^13^C) were observed in the subtending leaf, bract and capsule wall, suggesting that carbon assimilation of three organs was restricted and the limitation was most pronounced in the subtending leaf. Changes in the activities of sucrose phosphate synthase (SPS), sucrose synthase (SuSy), invertases as well as the reduction in expression of sucrose transporter (*GhSUT1*) led to variabilities in the sucrose content of three organs. Moreover, photosynthate distribution from subtending leaf to seeds plus fibers (the components of boll weight) was significantly restricted and the photosynthetic contribution rate of subtending leaf to boll weight was decreased, while contributions of bracts and capsule wall were increased by drought. This, in conjunction with the observed decreases in boll weight, indicated that the subtending leaf was the most sensitive photosynthetic organ to drought and was a dominant driver of boll weight loss under drought. Therefore, the subtending leaf governs boll weight loss under drought due to limitations in carbon assimilation, perturbations in sucrose metabolism and inhibition of sucrose transport.

## Introduction

Cotton (*Gossypium hirsutum*) is the most valuable fiber crop around the world (Ai et al., 2017), and drought represents one of the greatest threats to cotton production globally (Abdelraheem et al., 2019). Lint yield is often expressed as the product of the following yield components: boll weight, boll number and lint percentage. Although drought primarily limits yield owing to reductions in boll number per plant, average boll mass can also be an important contributor to drought-induced reductions in lint yield (Sharma et al., 2015; Hu et al., 2018). Photosynthetic carbon assimilation is a prerequisite for production of cotton boll biomass (Wang et al., 2019), and specifically in cotton, each subtending leaf with corresponding boll make up a distinctive “source-sink system” (Wang et al., 2021), as the subtending leaf is known to provide about 60% of the total photosynthate required for boll development (Wullschleger and Oosterhuis, 1990). Nevertheless, in this system, the bracts and capsule wall are also green tissues that exhibit relatively high photosynthetic rates (Elmore, 1973). The photosynthetic rate per unit area of bract and capsule wall can reach 20.4-26.3% and 60.3-72.8% of that of the subtending leaf, respectively (Zhan et al., 2014). Hu et al. (2012) observed that the relative contribution of cotton bracts plus capsule wall photosynthesis to boll weight can reach more than 24% under normal growing conditions. Since the structure of these photosynthetically active organs is different, their response to abiotic stresses is not the same (Hu et al., 2014), and so their individual contributions to boll weight may vary under abiotic stress conditions.

Sucrose is not only one of the most important photosynthetic products and subsequent carbon metabolism material, but it is also the primary photosynthate delivered from photosynthetic organs to other sink organs. Hence, sucrose metabolism is considered an important factor that governs sink strength and the development of seed and fiber weight in growing cotton bolls (Pugh et al., 2010). In photosynthetic carbon assimilation, several enzymes have been identified to play critical roles in determining source-sink balance. Ribulose-1,5-bisphosphate carboxylase (Rubisco, E.C. 4.1.1.39) (Dean et al., 1989) catalyzes the carboxylation step of the Calvin cycle and the production of 3-phosphoglycerate (Parry et al., 2002), while sucrose phosphate synthase (SPS, E.C. 2.4.1.14), and sucrose synthase (SuSy, E.C. 2.4.1.13) play direct roles in sucrose accumulation (Hendrix and Huber, 1986). As SPS catalyzes the synthesis of sucrose phosphate from 6-P fructose and uridine diphosphate glucose (UDPG), while SuSy has a catalytic function in the synthesis of sucrose from UDPG and fructose (Liu et al., 2013). Whereas, the soluble acid and alkaline invertases (E.C. 3.2.1.26) catalyze irreversibly sucrose hydrolysis into glucose and fructose (Kaur et al., 2007). The export of sucrose from photosynthetic organs is mainly regulated by proteins including the Sugars Will Eventually Be Exported Transporters (SWEET protein) and Sucrose Transporters/Carriers (SUT/SUC protein) (Ding et al., 2021). Specifically, SWEET proteins are involved in sucrose loading in the phloem (Chen et al., 2015), as these proteins first transport sucrose into the apoplast (Baker et al., 2012; Braun, 2012) and subsequently, sucrose is transferred by SUT protein into the sieve-companion cell (Sauer, 2007). Previous research has suggested that altered expression of *SWEET* or *SUT* genes would affect the export of sucrose and consequently the development of non-photosynthetic organs, especially under unfavorable conditions (Klemens et al., 2013). Taking in consideration that both carbon assimilation and transport are known to be sensitive to environmental conditions (Wang et al., 2017), it is safe to assume that abiotic stresses that affect carbon metabolism or transport, such as water deficit, will affect cotton boll development and weight.

As it is well known, adequate water supply is essential for plant growth and boll development (Gao et al., 2020). However, due to climate change, the frequency and intensity of drought events are increasing, which cause a major threat to cotton productivity worldwide (Bahrami et al., 2014). Previous reports have shown that drought decreases cotton boll weight (Dağdelen et al., 2009; Lokhande and Reddy, 2014), and these declines were closely related to reductions in the photosynthetic rates of major source organs (Zahoor et al., 2017b). Drought has been shown to inhibit photosynthesis by limiting the Calvin cycle, down-regulating Ribulose bisphosphate carboxylase small chain (*RBCS*) expression, and decreasing Rubisco activity (Zahoor et al., 2017a; Gao et al., 2021). Additionally, other photosynthetically active tissues such as cotton bracts and capsule wall are sensitive to low soil moisture, as evidenced by reductions in chlorophyll content and net photosynthetic rate (*Pn*) of these tissues under water deficit (Hu et al., 2013; Zhan et al., 2014). Previous studies on the impacts of drought on photosynthesis and carbon metabolism mainly focused on mainstem and subtending leaves (Chastain et al., 2014; Chastain et al., 2016; Zhao et al., 2019); however, studies focused on the effects of drought on carbon metabolism of bracts and capsule wall are lacking. Cotton cultivars with varying drought sensitivity exhibit various responses to drought stress (Riaz et al., 2013), as evidenced by numerous physiological mechanisms, including leaf photosynthesis and carbohydrate metabolism (Parida et al., 2007; Sicher et al., 2015). However, the specific responses of carbon metabolism of cotton photosynthetic organ to drought among different cultivars need further research.

Therefore, it is essential to systematically investigate and compare the production and distribution of carbon in the subtending leaf, bracts, and capsule wall and explore which photosynthetic organ mainly affects boll weight formation under water-deficit stress at the flowering and boll formation stages. It was hypothesized: (1) carbon metabolism of subtending leaf, bracts and capsule wall and their relative contribution to boll mass differ under drought conditions, and (2) the responses mechanisms of carbon metabolism in different cultivars to drought exhibit variously. The objectives were: (1) to compare changes in carbon metabolism among three photosynthetic organs under drought, (2) to determine the relative contribution to boll weight of each photosynthetic organ, and (3) to evaluate the differences in the response mechanisms of cultivars with different drought sensitivity to drought.

## Materials and methods

### Plant materials and experimental design

Pot experiments were performed out in a greenhouse with a retractable transparent plastic top and walls in 2018 and 2019 at Pailou Experimental Station, Nanjing Agricultural University, Nanjing (118°50′E, 32°02′N), China. The weather data during the experimental period was presented in **Supplementary** **Figure 1**. The daily maximum temperature and daily minimum temperature from July 1 to August 31 were 33.3 °C and 26.0 °C in 2018, 32.8 °C and 24.9 °C in 2019, and the average sunshine duration was 7.6 h and 6.0 h in 2018 and 2019, respectively. The cotton cultivars selected for the experiment were Dexiamian 1 and Yuzaomian 9110, with a similar individual boll growth period of about 38 days, as reported in our previous experiment (Zou et al., 2020). On 7 April 2018 and 12 April 2019, seeds were sown in a nursery bed. Uniform seedlings with three true leaves were transplanted in 13 L plastic pots, which were filled with 12 kg clay soil containing 1.1 and 1.2 g kg^-1^ total nitrogen (N), 65.1 and 71.3 mg kg^-1^ available N, 127.9 and 138.7 mg kg^-1^ available potassium (K),21.5 and 22.5 mg kg^-1^ available phosphorus (P), 17.8 and 18.3 g kg^-1^ organic matter in 2018 and 2019. A single plant was planted in each pot, and each pot was considered as a replication. Each treatment consisted of 58 pots, and each pot was evenly fertilized with 0.64 g P_2_O_5_ pot^-1^, 1.28 g K_2_O pot^-1^ and 2.78 g N pot^-1^ annually.

**Figure 1:**
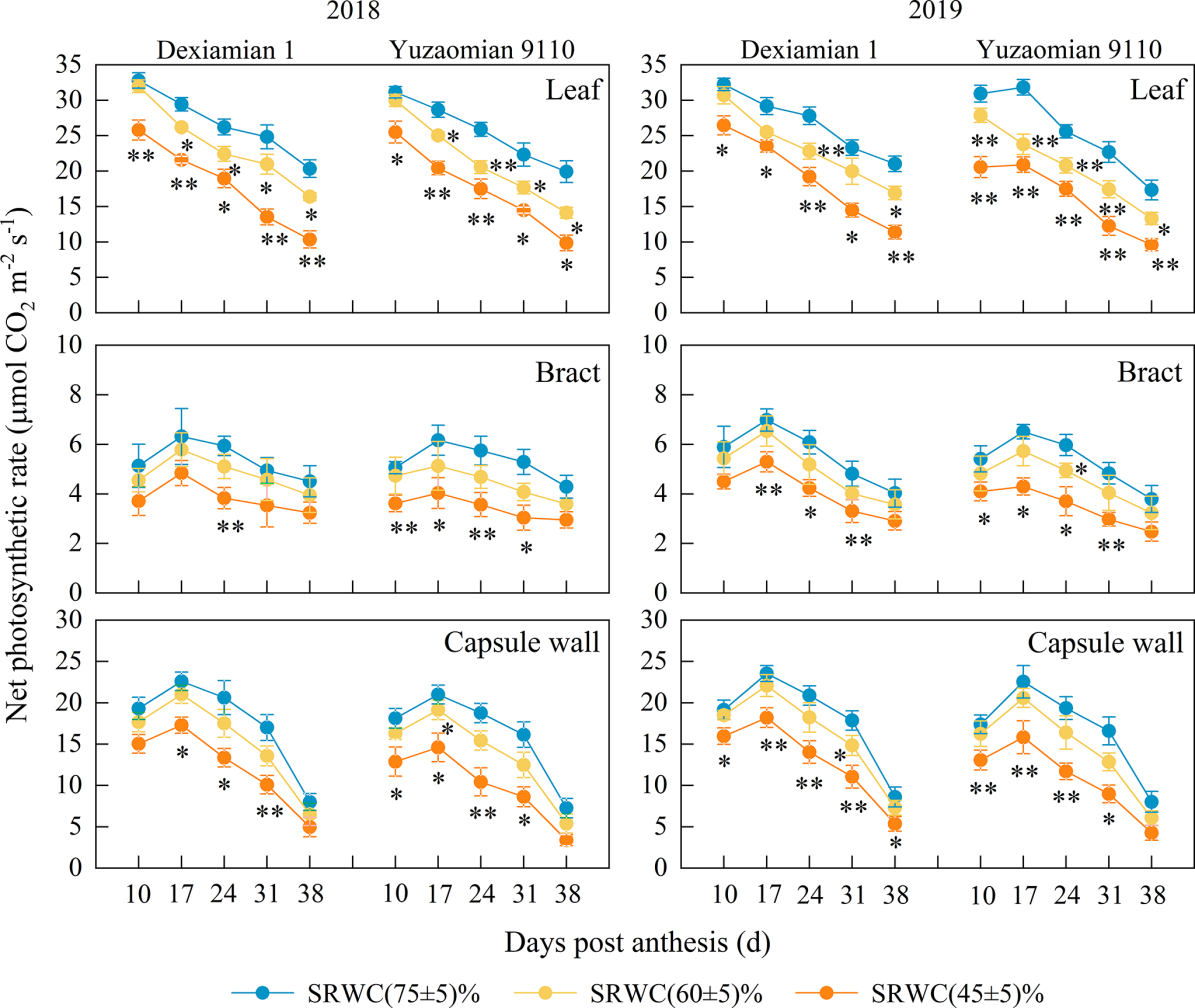
Effects of drought on the net photosynthetic rate (*Pn*) of the subtending leaf, bract and capsule wall in 2018 and 2019. Vertical bars denote standard error (n = 3). The asterisks indicate significantly differences between SRWC(75 ± 5)% and SRWC(60 ± 5)% or SRWC(45 ± 5)% within each cultivar for a t-test (* *P*< 0.05, ** *P*< 0.01).

All plants were well-watered to keep the soil relative water content (SRWC) at (75 ± 5)% until roughly 50% of the flowers on the first fruiting position on the 4-6th fruiting branches above the cotyledon node opened, which were started on 24 June 2018 and 30 June 2019. Then, three soil water treatments consisting of control (SRWC at 75 ± 5%), moderate drought (SRWC at 60 ± 5%) and severe drought (SRWC at 45 ± 5%) were established according to previous studies (Wang et al., 2016b). Soil water content was determined following the method described by Liu et al. (2008). Soil samples from 0 to 20 cm depth were collected during soil water treatment from 1800 to 1900 h with a punch (2 cm diameter) from different pots of each treatment and then composite samples were collected. Fresh weight of the samples was determined and then these samples were oven-dried at 105°C for 8 h. Soil water content was expressed as g water g^-1^ dried soil. Cotton plants would be watered to the upper soil water limit (SRWC of 80%, 65%, 50%) in the next early morning, when SRWC of each treatment was smaller than the lower limit (SRWC of 70%, 55%, 40%). SRWC was measured every 2-3 days, and its trends in this study are shown in **Supplementary Figure 2**. The water treatments lasted until boll opening, 38 days, meaning that the number of days of drought corresponded to the days post anthesis (DPA) for the flowers of the first fruiting node on the 4-6th fruiting branches. On the day of establishment of different treatments, the date of flowering for the previously-noted fruiting branches was marked for subsequent sampling.

### Plant sampling

About eight marked cotton bolls with subtending leaves and bracts for each treatment were sampled on 10, 17, 24, 31 and 38 DPA at 0900-1000 h. By severing the main vein, the subtending leaves were rinsed with distilled water, then separated into two halves. The bracts were also washed with distilled water. The capsule walls were removed from the boll with sterile tweezers on ice. Half of the samples (subtending leaves, bracts and capsule walls) were immediately placed in liquid-nitrogen, then stored in an ultralow temperature freezer (-80 °C) for following analysis, the other half were dried at 105 °C for 30 min, then by 72 h at 70 °C to achieve a constant weight.

### Determination of chlorophyll content and Pn

Total chlorophyll content in the three subtending leaves, bracts, and capsule walls for each treatment at 10, 17, 24, 31 and 38 DPA was measured using the ethanol and acetone extraction colorimetric method according to Moran (1982). The *Pn* of above photosynthetic organs from three plants per treatment was determined from 0900 to 1100 h at 10, 17, 24, 31 and 38 DPA using a portable photosynthesis system (Li-6400, Li-COR, Lincoln, NE, USA). A fluorometer leaf chamber (6400-40) was used for measuring *Pn* of the subtending leaf and bract according to the following settings: 380 ± 5 μmol mol^-1^ CO_2_ concentration, 1500 μmol m^-2^ s^-1^ photosynthetic photon flux density (red and blue light sources), (65 ± 5)% air relative humidity. A conifer chamber (6400-05) was used for measuring the *Pn* of the entire boll following the same settings except that the red and blue light sources were replaced by white light.

### Sucrose content and enzymatic analyses

Dried samples of the subtending leaf, bract or capsule wall were ground to a fine powder, then 0.15 g were added to 5 ml ethanol that was 80 percent (v/v). The mixtures were exposed in an 80 °C water bath for 40 min before being filtered. The foregoing procedure was performed three times, and the three supernatants were mixed and diluted in 80% ethanol to make 25 ml. The sucrose content was determined with the supernatant according to Hendrix (1993).

Rubisco was obtained from frozen samples in accordance with Hu et al. (2015), then the obtained extract (5 μl) was added to 985 μl of reaction solution as described by Zahoor et al. (2017a). Then, to drive the beginning of the reaction, appended 50 mM ribulose-1,5-bisphosphate for 10 μl. The activity was detected through detecting the oxidation rate of nicotinamide adenine dinucleotide over 1 min at 340 nm at 25°C.

Frozen samples (0.3 g) were used to extract the SPS and SuSy, which were ground in cooled extraction solution (5 ml) containing 10 mM MgCl_2_, 1 mM phenylmethylsulfonyl fluoride, 2% polyvinylpyrrolidone, 1 mM ethylene glycol bis-(2-aminoethyl ether)-tetraacetic acid, 1% (v/v) Triton X-100, 1 mM ethylenediaminetetraacetic acid, 0.5% (w/v) bovine serum albumin, and 50 mM N-(2-hydroxyethyl) piperazine-N’-(2-ethane sulfonic acid)-NaOH buffer (pH 7.5). The supernatant was obtained after centrifuging the homogenate for 15 minutes at 15,000 ×g at 4°C. The SPS, SuSy activities were determined by evaluating the sucrose synthesis in accordance with Wang et al. (2017).

The soluble acid and alkaline invertases were extracted using the same method for extraction of SPS and SuSy, and measured with minor modifications as reported by Shu et al. (2009). The activity of acid invertase was assayed using the aforementioned extract (200 μl) with 2.3 ml reaction solution [1 M sucrose, 200 mM acetic acid-NaOH (pH 5.0)], then the alkaline invertase activity was determined using the extract (200 μl) with 2.3 ml reaction solution [200 mM sodium acetateacetic acid (pH 7.5), 1 M sucrose]. Then the remaining steps were consistent with Shu et al. (2009).

### Carbon isotope analysis

At 10 and 31 DPA, the subtending leaf, bract or capsule wall from different three plants of each treatment were exposed separately to a sealed, transparent chamber which contained ^13^CO_2_ (Shanghai Engineering Research Center of Stable Isotope, China) for 5 ml, for 4 h (08:30-12:30) with three replications. After 24 h (the next afternoon at 12:30), the isotope-labeled subtending leaves, with their corresponding bolls (including capsule walls, seeds and fibers) and bracts, the isotope-labeled bracts, with their corresponding bolls and subtending leaves, the isotope-labeled capsule walls with their corresponding subtending leaves, bracts, seeds and fibers were collected to estimate carbon isotope composition (δ^13^C), using an EA-1110 elemental analyzer (Carlo Erba Thermoquest, Milan, Italy) at 1020°C coupled to an isotope ratio mass spectrometer (Finnigan MAT, Bremen, Germany). Carbon isotopic value was expressed in δ^13^C(‰), and was calculated as:


(1) $$\delta^{13}C\left(‰\right)=\left[\frac{{{(}^{13}C{/}^{12}C)}_{sample}}{{{(}^{13}C{/}^{12}C)}_{standard}}-1\right]\times 10$$

Where (^13^C/^12^C)_standard_ is the standard value of the Pee Dee Belemnite for the isotope ratio, and (^13^C/^12^C)_standard_ is the sample value for the isotope ratio respectively. To estimate the amount of ^13^C added by labelling, δ notation is expressed in atom% as follows:


(2) $$atom\%=\frac{\left(\delta^{13}C+1000\right)\times R_{PDB}}{\left(\delta^{13}C+1000\right)\times R_{PDB}+1000}\times 100$$

Where R_PDB_ (0.00112372) is the (^13^C/^12^C)_standard_ of the Pee Dee Belemnite.> To calculate the ^13^C content of the samples, we used the equation referring to Ruehr et al. (2009):


(3) $${\;}^{13}C\left(mg\right)=\frac{atom{\%}_{s}-atom{\%}_{n}}{100}\times w\times \frac{C\%}{100}$$

Where ^13^C content (expressed as mg) is the total amount of ^13^C in the organs, atom%_s_ is the atom% of the labelled sample, atom%_n_ is the atom% of the unlabeled natural sample, w is the dry weight of organ biomass (expressed as mg dry biomass), and C% is the percentage of total C in the sample. ^13^C allocation proportion of each organ was calculated based on ^13^C content, and mathematically expressed as:


(4) $${\;}^{13}C\;allocation\;proportion\left(\%\right)=\frac{{\;}^{13}C{\;}_{i}}{{\;}^{13}C{\;}_{total}}\times 100$$

Where ^13^C_i_ is the ^13^C content of the organ component, and ^13^C_total_ is the sum ^13^C content of the source-sink system including subtending leaf, bracts, and boll (capsule walls, seeds and fibers).

### Relative contribution to boll weight of photosynthetic organs

The contributions to boll weight of different photosynthetic organs were determined using the modified approach published by Araus et al. (1993). From 10 DPA to 38 DPA, aluminum foil was used to cover the subtending leaves, bracts, and capsule walls, respectively, from three different plants per treatment. To allow organs for gas exchange, a needle was used to create small holes in the foil. The biomass of seed and fiber per boll was assessed at 38 DPA of subtending leaf-darkened, bract-darkened or capsule wall-darkened plants. The relative contributions of the subtending leaf, bract and capsule wall to boll weight were computed with the following equation:


(5) $$Relative\;contribution\;\left(\%\right)=\frac{\left(control\;weight-darkened\;weight\right)}{control\;weight}\times 100$$

### Measurement of seed cotton yield

At plant maturity, all open bolls on the nine plants for each treatment were counted and harvested by hand to determine boll number per plant. Thereafter, samples were transferred to the lab, where they were weighed to obtain a total seed cotton yield per plant and an average boll weight.

### Quantitative real-time PCR

The samples of subtending leaf, bract and capsule wall were used for quantitative real-time PCR (qRT-RCR) with two cultivars, Dexiamian 1 and Yuzaomian 9110; two water treatments, control [SRWC(75 ± 5)%] and 10-day, 31-day drought [SRWC(45 ± 5)%]; and three biological replicates per treatment at 0900-1000 h in 2019. The Plant Total RNA Extraction Kit was used to extract total RNA in the samples (TIANGEN, Beijing, China). RNA purity was determined using NanoDrop ultraviolet spectrophotometer (Nanodrop Technologies, Wilmington, DE, USA), and RNA fragment length or integrity was detected using Agilent 2100 Bioanalyzer (Agilent Technologies, Santa Clara, CA, USA). Primer Premier 5.0 was used to design the primers, which were then produced commercially by Invitrogen Corporation (Beijing, China) (**Supplementary** **Table 1**). PrimeScript RT reagent Kit with gDNA Eraser (TaKaRa, Kusatsu, Japan) was utilized to make first-strand cDNA, then qRT-PCR was done using an Applied Biosystem 7500 real-time PCR system. The program was set as follows: initial denaturation at 95 °C, 30 s, followed by denaturation with 40 cycles at 95 °C, 5 s, then collecting fluorescence at 60 °C, 40 s, to establish the melting curve, use 95 °C, 10 s, 60 °C, 60 s, 95 °C, 15 s after the amplification, and slowly heated from 60 °C to 99 °C. Internal control 18S rRNA (L24145) was used to quantify and standardize gene expression (Wang et al., 2017).

**Table 1:** Effects of soil relative water content (SRWC) on seed cotton yield components in 2018 and 2019.

Cultivar	Soil relative water content SRWC(%)	2018			2019		
		Boll number	Boll weight	Seed cotton yield	Boll number	Boll weight	Seed cotton yield
		(no. plant^-1^)	(g)	(g plant^-1^)	(no. plant^-1^)	(g)	(g plant^-1^)
Dexiamian 1	75 ± 5	10.7 ± 0.3 a	4.9 ± 0.1 a	51.7 ± 1.3 a	10.3 ± 0.3 a	5.0 ± 0.1 a	51.4 ± 0.7 a
	60 ± 5	8.0 ± 0.3 b	4.0 ± 0.1 b	32.1 ± 0.6 b	7.7 ± 0.2 b	4.1 ± 0.1 b	31.6 ± 1.2 b
	45 ± 5	6.3 ± 0.4 c	3.8 ± 0.1 b	23.9 ± 0.9 c	6.0 ± 0.4 c	3.9 ± 0.2 b	22.9 ± 0.5 c
Yuzaomian 9110	75 ± 5	10.3 ± 0.2 a	4.8 ± 0.1 a	50.1 ± 1.3 a	10.3 ± 0.2 a	4.7 ± 0.1 a	48.6 ± 1.2 a
	60 ± 5	7.4 ± 0.2 b	3.8 ± 0.1 b	28.3 ± 0.4 b	7.2 ± 0.1 b	3.8 ± 0.1 b	27.1 ± 0.2 b
	45 ± 5	5.3 ± 0.2 c	3.2 ± 0.1 c	17.1 ± 0.4 c	5.2 ± 0.3 c	3.1 ± 0.1 c	16.1 ± 0.4 c
Significance of factors							
Cultivar		*	**	**	NS	**	**
SRWC		**	**	**	**	**	**
Cultivar × SRWC		NS	**	*	NS	*	NS

Data are expressed as means ± SE, n = 9. Two-way analysis of variance (ANOVA) was conducted to compare the difference with the Duncan’s multiple range test. Different letters indicate significant differences within each cultivar at P< 0.05. * and ** mean significant differences at P< 0.05 and P< 0.01, respectively; NS means non-significant differences.

### Statistical analysis

All parameters were measured for a minimum of three biological replicates and analyzed with the SPSS Statistics 26.0 (IBM, Armonk, NY, USA). The impacts of water treatment, cultivar, and their interaction for yield components were investigated using a two-way analysis of variance (ANOVA) following Duncan’s multiple range test, different lower-case letters indicate significantly difference at *P*< 0.05; and other indicators with comparison in two groups were analyzed by using Student’s t-test. * and ** represent *P<* 0.05 and *P*< 0.01. Figures were designed by Origin 2019 (OriginLab, Northampton, MA, USA).

## Results

### Seed cotton yield in response to drought

The number of bolls per plant, boll weight, seed cotton yield under drought were considerably lower than that of well-watered plants (control; **Table 1**). Noticeable disparities were uncovered in the above indicators between two cultivars. In 2018-2019, compared to control, under SRWC(60 ± 5)% and SRWC(45 ± 5)%, boll weight for Dexiamian 1 decreased by 17.0-17.5% and 21.4-22.1%, respectively. Furthermore, boll weight for Yuzaomian 9110 decreased by 20.0-21.3% and 33.2-33.4%, respectively.

### Carbon assimilation of different photosynthetic organs in response to drought

Compared with the control, chlorophyll content of the subtending leaf was significantly decreased under SRWC(60 ± 5)% and SRWC(45 ± 5)% (**Supplementary Figure 3**). However, the chlorophyll content of the bract did not alter significantly amongst the soil water deficit treatments. Compared with the control, chlorophyll content of the capsule wall was significantly decreased under SRWC(45 ± 5)% at 24, 31 DPA. The average *Pn* of subtending leaf in two years was reduced by 3.6-19.5% and 19.6-47.5% under SRWC(60 ± 5)% and SRWC(45 ± 5)% for Dexiamian 1; and by 6.8-26.2% and 25.8-50.5% for Yuzaomian 9110, from 10-38 DPA (**Figure 1**). There were little significant differences in *Pn* between SRWC(60 ± 5)% and the control for the bract or capsule wall in either cultivar; however, a significant difference in *Pn* between SRWC(45 ± 5)% and the control was observed for the bract and capsule wall prior to 38 DPA, and the average *Pn* of bract and capsule wall in two years was reduced by 24.1-32.9% and 19.4-38.8% under SRWC(45 ± 5)% for Dexiamian 1, and by 26.4-40.4% and 26.9-46.2% for Yuzaomian 9110, respectively, from 10-31 DPA. The chlorophyll content and *Pn* under SRWC(60 ± 5)% decreased significantly only in the subtending leaf, but not in the bract and capsule wall, suggesting that the subtending leaf might be more sensitive to moderate drought than the other two photosynthetic organs. While the *Pn* under SRWC(45 ± 5)% decreased markedly in all three photosynthetic organs. Therefore, in order to reveal and compare the relative contribution of photosynthesis by different photosynthetic organs to boll weight under drought, we focused our efforts on the SRWC(45 ± 5)% treatment.

**Figure 2:**
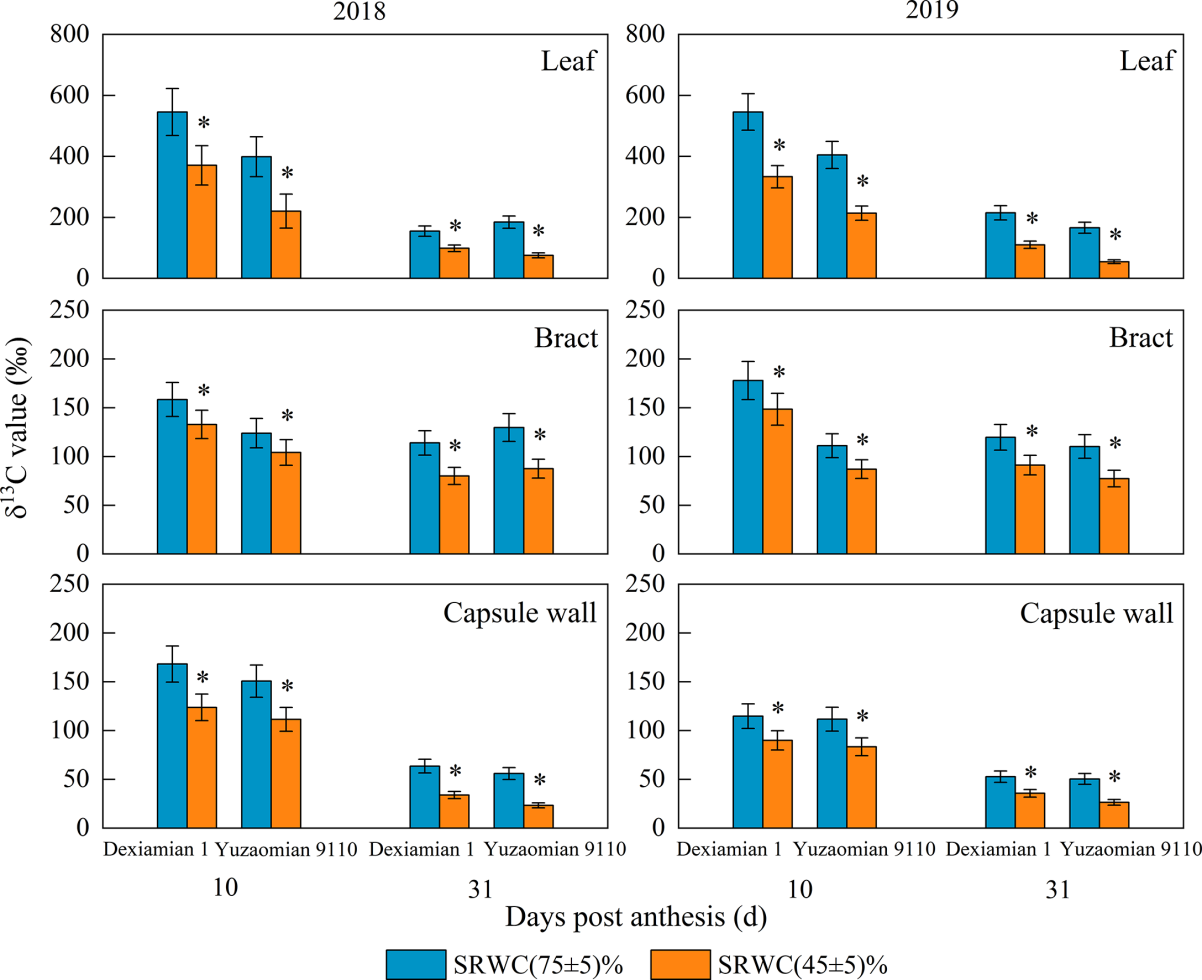
Effects of drought on the δ^13^C value of the subtending leaf, bract and capsule wall in 2018 and 2019. Vertical bars denote standard error (n = 3). The asterisks indicate significantly differences between SRWC(75 ± 5)% and SRWC(60 ± 5)% or SRWC(45 ± 5)% within each cultivar for a t-test (* *P*< 0.05).

The δ^13^C values of the subtending leaf, bract and capsule wall at 10 and 31 DPA of two years were decreased under SRWC(45 ± 5)% (**Figure 2**), and the decrease in the subtending leaf was greater than the bract and capsule wall. In addition, the reduction of δ^13^C value in the three photosynthetic organs under SRWC(45 ± 5)% of Yuzaomian 9110 was more significant than that of Dexiamian 1.

**Figure 3:**
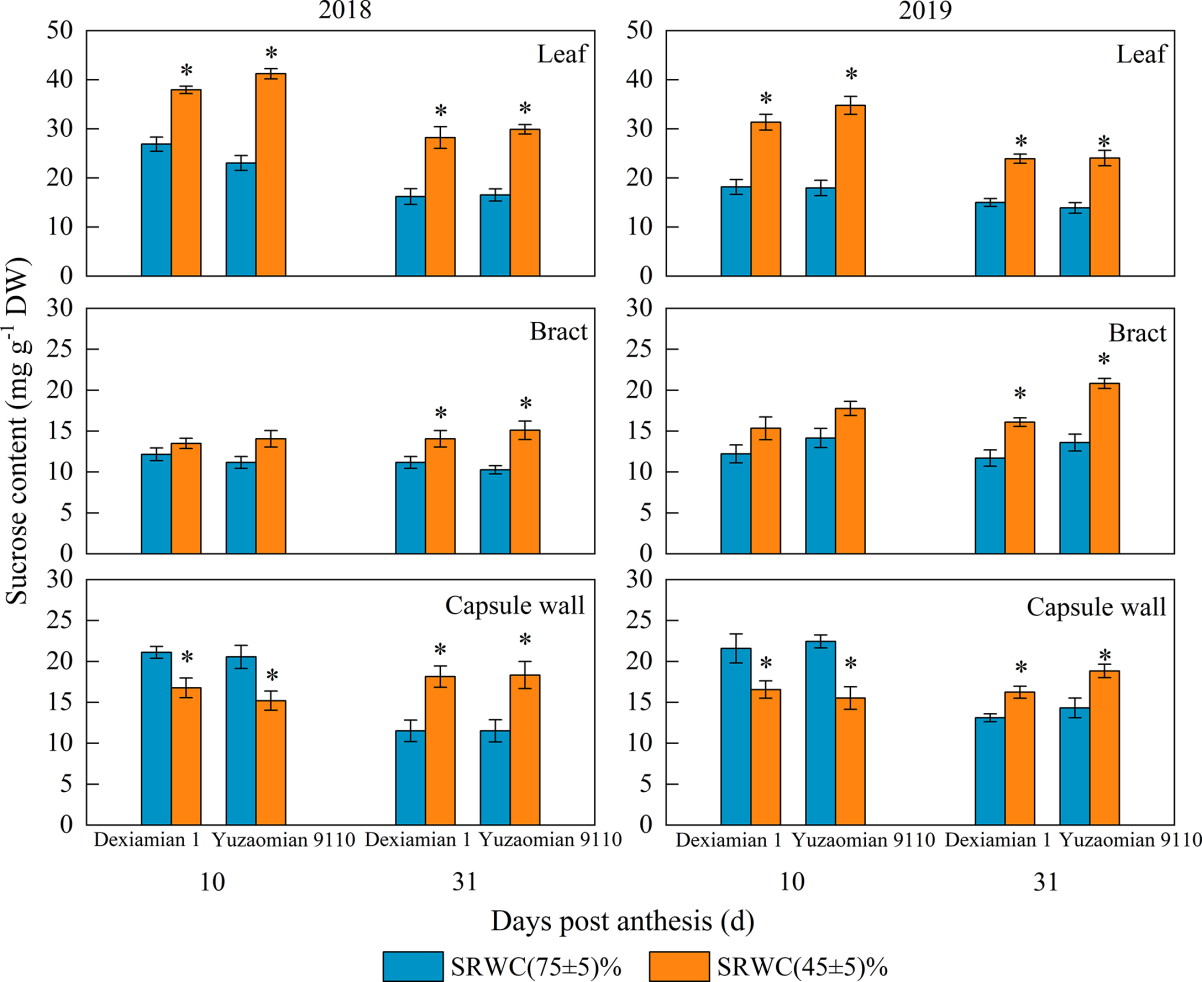
Effects of drought on the sucrose content of the subtending leaf, bract and capsule wall in 2018 and 2019. Vertical bars denote standard error (n = 3). The asterisk indicates significant differences between SRWC(75 ± 5)% and SRWC(45 ± 5)% within each cultivar for a t-test (*P*< 0.05).

Under SRWC(45 ± 5)% at 10 DPA, the average sucrose content in two years increased by 35.6% for Dexiamian 1 and 46.2% for Yuzaomian 9110 in the subtending leaf (**Figure 3**); and decreased by 28.1% for Dexiamian 1 and 39.9% for Yuzaomian 9110 in the capsule wall, relative to well-watered conditions. However, the sucrose content of the bract did not alter significantly between the control and SRWC(45 ± 5)%. Under SRWC(45 ± 5)% at 31 DPA, the sucrose contents of three photosynthetic organs in two years were increased, and the increase in the subtending leaf was greater than the bract and capsule wall. Moreover, Yuzaomian 9110 was more affected than Dexiamian 1, considering the sucrose contents of three photosynthetic organs under SRWC(45 ± 5)%.

**Figure 4:**
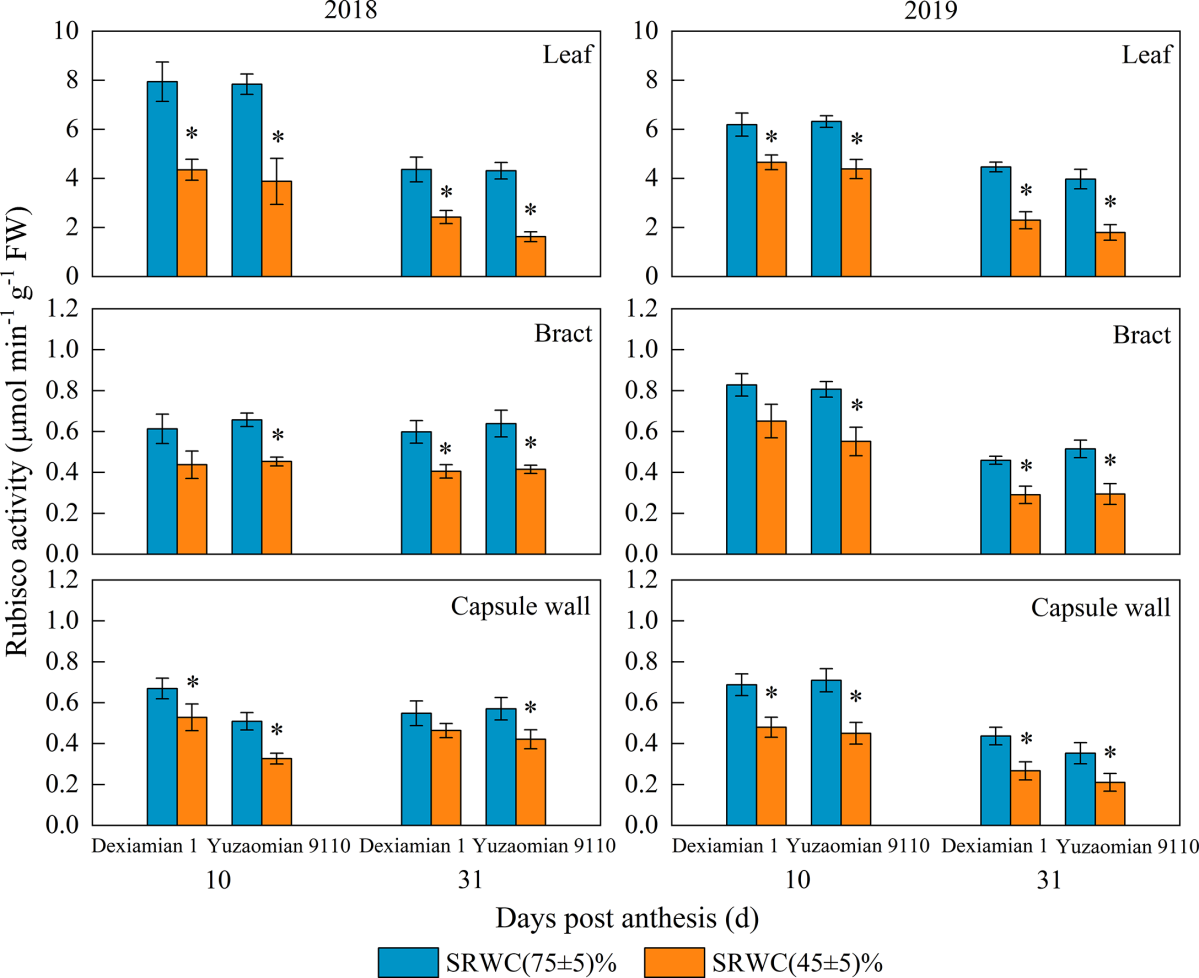
Effects of drought on ribulose-1,5-bisphosphate carboxylase (Rubisco) activity of the subtending leaf, bract and capsule wall in 2018 and 2019. Vertical bars denote standard error (n = 3). The asterisk indicates significant differences between SRWC(75 ± 5)% and SRWC(45 ± 5)% within each cultivar for a t-test (*P*< 0.05).

**Figure 5:**
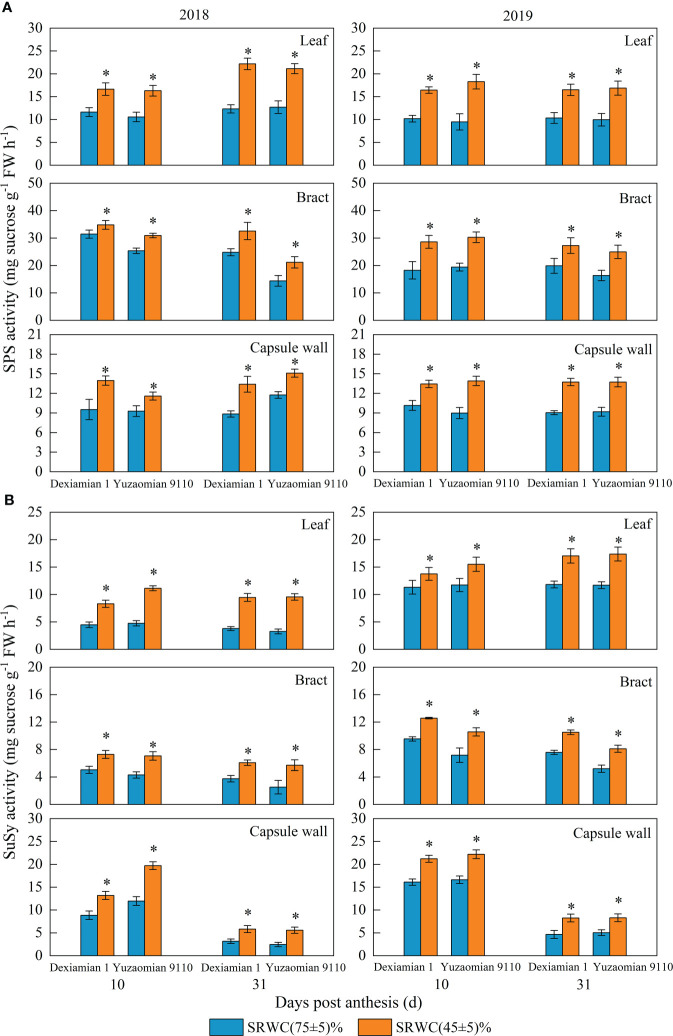
Effects of drought on the activities of sucrose phosphate synthase (SPS) **(A)** and sucrose synthase (SuSy) **(B)** of the subtending leaf, bract and capsule wall in 2018 and 2019. Vertical bars denote standard error (n = 3). The asterisk indicates significant differences between SRWC(75 ± 5)% and SRWC(45 ± 5)% within each cultivar for a t-test (*P*< 0.05).

**Figure 6:**
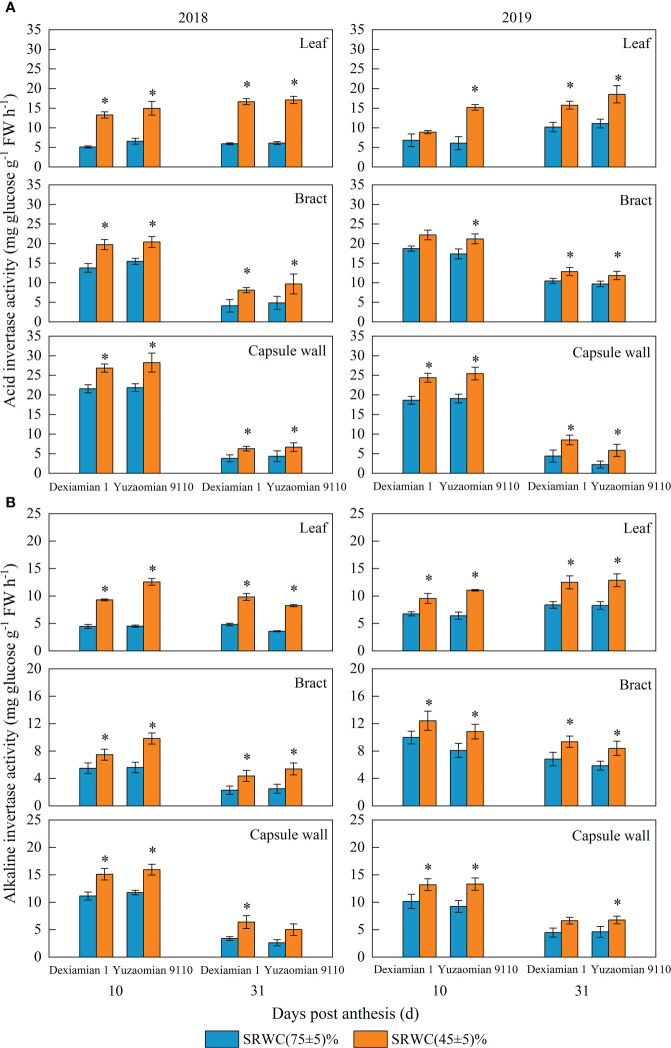
Effects of drought on the activities of the acid **(A)** and alkaline **(B)** invertase of the subtending leaf, bract and capsule wall in 2018 and 2019. Vertical bars denote standard error (n = 3). The asterisk indicates significant differences between SRWC(75 ± 5)% and SRWC(45 ± 5)% within each cultivar for a t-test (*P*< 0.05).

Compared with the well-watered control, Rubisco activities of the aforementioned photosynthetic organs were decreased under SRWC(45 ± 5)% for both cultivars (**Figure 4**). The activities of SPS, SuSy, the acid and alkaline invertase in the three photosynthetic organs were obviously increased at 10 and 31 DPA under SRWC(45 ± 5)% for both cultivars, compared to the control (**Figures 5**, **6**). The amplitude of variation in the subtending leaf was greater than the bract and capsule wall, and compared to Dexiamian 1, Yuzaomian 9110 was relatively sensitive to SRWC(45 ± 5)% when it comes to the activities of SPS, SuSy, the acid and alkaline invertase.

### Distribution of carbon assimilation products from different photosynthetic organs under drought

**Figure 7:**
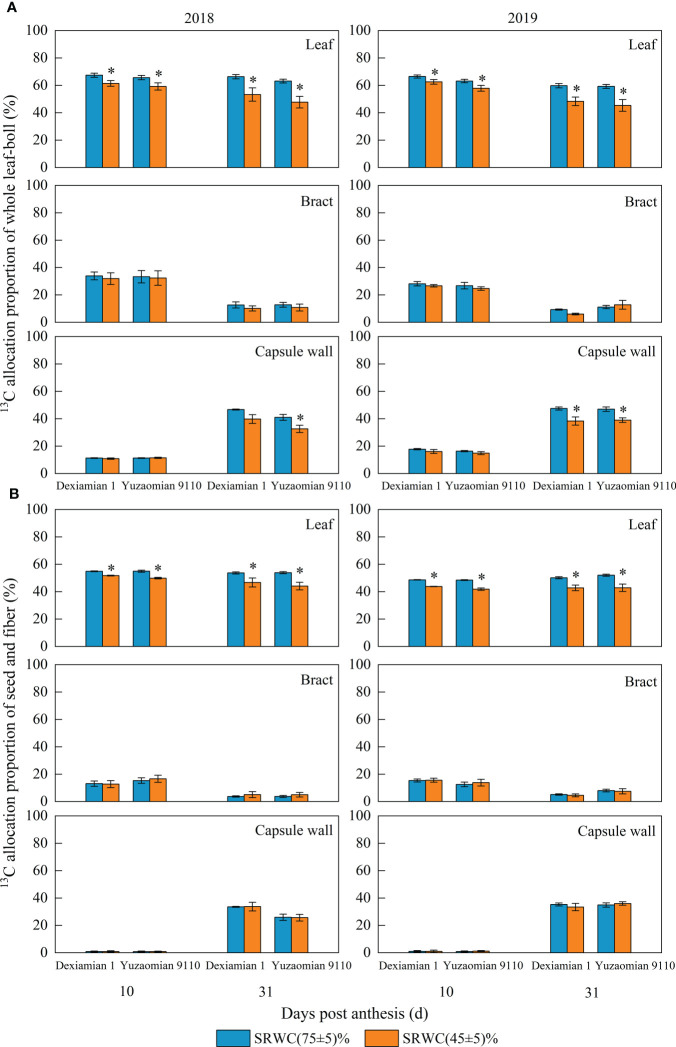
Effects of drought on ^13^C allocation proportion of whole leaf-boll **(A)** and seed-fiber **(B)** after different photosynthetic organs (subtending leaf, bract and capsule wall) were fed with ^13^CO_2_ in 2018 and 2019. Leaf, bract and capsule wall of A stand for [^13^C content in corresponding bract plus boll (including capsule wall, seed and fiber)]/(^13^C content in total organs) × 100%, [^13^C content in corresponding subtending leaf plus boll (capsule wall, seed, fiber)]/(^13^C content in total organs) × 100%, (^13^C content in corresponding subtending leaf, bract, seed plus fiber)/(^13^C content in total organs) × 100% after feeding the subtending leaf, bract and capsule wall with ^13^CO_2_, respectively. Leaf, bract and capsule wall of B stand for (^13^C content in corresponding seed plus fiber)/(^13^C content in total organs) × 100% after feeding the subtending leaf, bract and capsule wall with ^13^CO_2_, respectively. Total organs included subtending leaf, bract, capsule wall, seed and fiber. Vertical bars denote standard error (n = 3). The asterisk indicates significant differences between SRWC(75 ± 5)% and SRWC(45 ± 5)% within each cultivar for a t-test (*P*< 0.05).

After feeding the subtending leaf with ^13^CO_2_ at 10 and 31 DPA, a notable decrease of ^13^C allocation proportion in the corresponding bract plus boll (including capsule wall, seed and fiber) was detected under severe drought (**Figure 7A**), and the average ^13^C allocation proportion of two years was decreased by 7.4% and 20.5% for Dexiamian 1, and 9.1% and 23.9% for Yuzaomian 9110, respectively, compared to the control; however, there was no significant difference in the ^13^C allocation proportion in the corresponding subtending leaf plus boll between SRWC(45 ± 5)% and the control after feeding the bract with ^13^CO_2_. After feeding the capsule wall with ^13^CO_2_, the ^13^C allocation proportion in the corresponding subtending leaf, bract, seed plus fiber was markedly decreased at 31 DPA under SRWC(45 ± 5)%, compared with the control.

Additionally, after feeding the subtending leaf with ^13^CO_2_ at 10 and 31 DPA, ^13^C allocation proportion in the corresponding seed plus fiber was decreased under SRWC(45 ± 5)% (**Figure 7B**), and the average ^13^C allocation proportion of two years was decreased by 7.8% and 15.0% for Dexiamian 1, and 11.5% and 17.9% for Yuzaomian 9110, respectively, compared to the control. Conversely, the ^13^C allocation proportion in the corresponding seed plus fiber was not decreased after feeding the bract or capsule wall with ^13^CO_2_.

**Figure 8:**
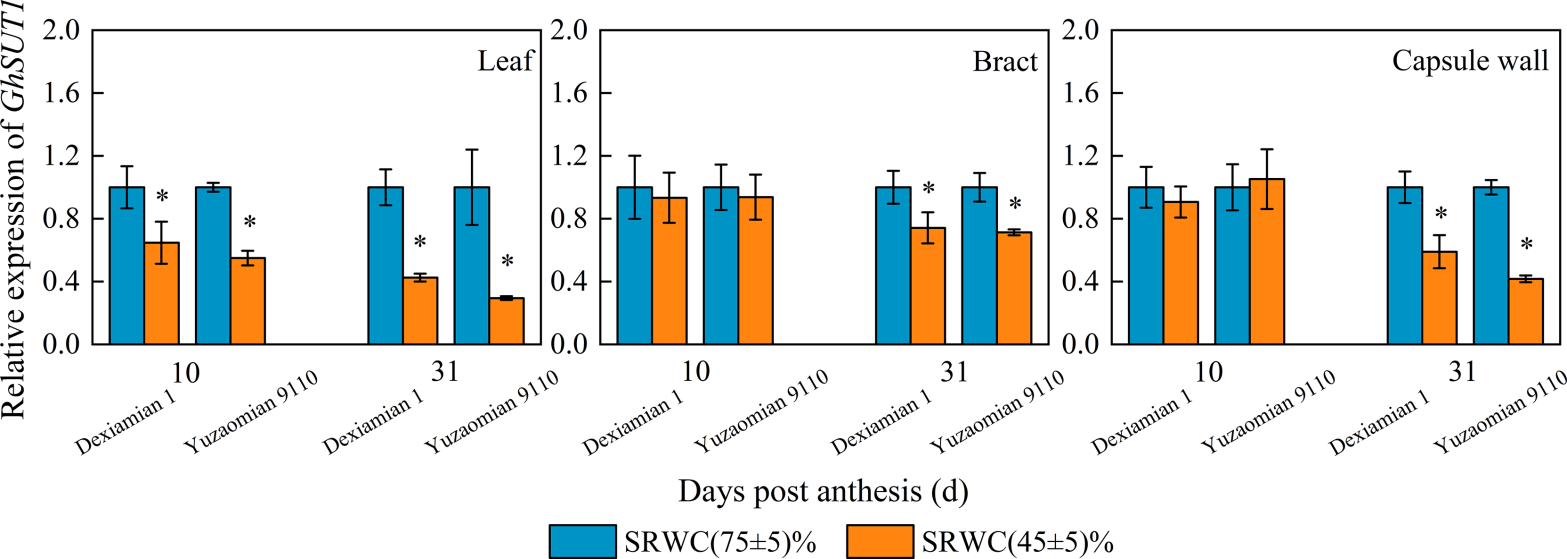
Effects of drought on the relative expression of sucrose transporter (*GhSUT1*) of the subtending leaf, bract and capsule wall in 2019. Vertical bars denote standard error (n = 6). The asterisk indicates significant differences between SRWC(75 ± 5)% and SRWC(45 ± 5)% within each cultivar for a t-test (*P*< 0.05).

The level of *GhSUT1* expression in the subtending leaf was reduced at 10 DPA and 31 DPA for both cultivars under SRWC(45 ± 5)%, compared to the well-watered plants (**Figure 8**), and the reduction in *GhSUT1* expression of Yuzaomian 9110 was larger than that of Dexiamian 1. *GhSUT1* expression did not alter significantly in the bract and capsule wall at 10 DPA, but a significant reduction was notable at 31 DPA under SRWC(45 ± 5)% compared to the control for both cultivars.

### Relative contribution of different photosynthetic organs to boll weight formation

**Figure 9:**
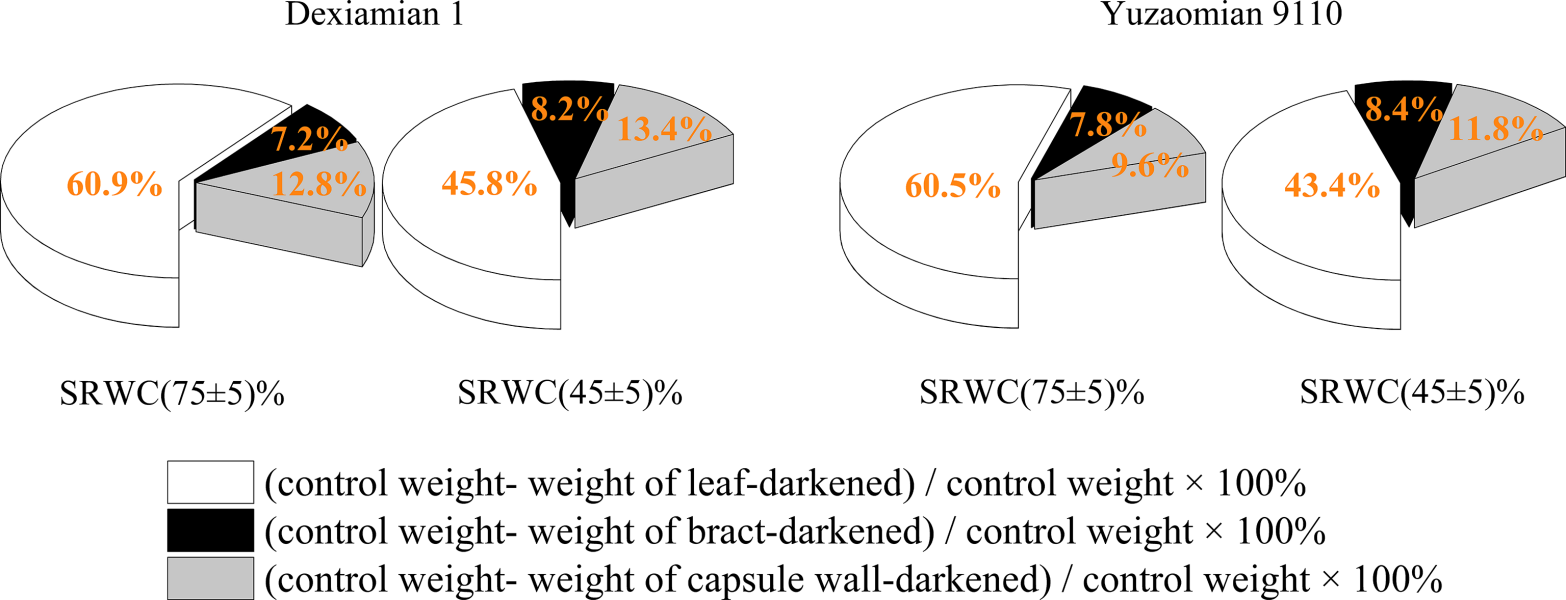
The relative contribution of different photosynthetic organs to cotton boll weight under drought in 2019.

Compared to well-watered plants, the relative contribution of the subtending leaf to boll weight decreased by 24.7% in Dexiamian 1 and 28.3% in Yuzaomian 9110 under severe drought (**Figure 9**), but the relative contributions of bract and capsule wall were increased in both cultivars.

## Discussion

### The influence of drought on the proportional contributions of various photosynthetic organs to boll weight

Previous studies have documented substantial declines in yield that were partially a function of boll weight reductions when drought stress was experienced at cotton boll formation stage (Wang et al., 2016b; Zou et al., 2020). Consistent with previous results, one of cotton yield components, boll weight under moderate and severe drought, were substantially lower than that of the well-watered in the present study. Furthermore, the decrease in boll weight for cotton cultivar (Yuzaomian 9110) was significantly greater than another cultivar (Dexiamian 1) (**Table 1**), which is in accordance with previous studies (Mehrabadi et al., 2015; Zou et al., 2020) where boll development of drought-sensitive cotton varieties was more susceptible to water stress compared with drought-tolerant cotton varieties. The carbohydrates required for boll filling are derived from leaves and other non-laminar photosynthetic tissues, each differing in their relative contribution to final boll mass (Simkin et al., 2019). Additionally, leaves, bracts and bolls each differ in their susceptibility to environmental stress (Hu et al., 2013). In this study, the relative photosynthetic contribution of the subtending leaf to boll weight was decreased by 24.7-28.3% under severe drought, while the relative contribution of bracts and capsule wall photosynthesis to boll weight was increased by 8.1-14.0% and 4.1-22.9% under severe drought (**Figure 9**). Despite those increases, boll weight was significantly decreased under drought, implying the lower photosynthetic rates of the subtending leaf and consequently, its reduced contribution might be the main driver of the observed reductions in boll weight under drought stress. Similar to our results, Zhang et al. (2011), in experiments with wheat (*Triticum aestivum* L.), reported that non-leaf organ photosynthetic contributions to yield not only rose with declining supply of water but were even higher than contributor of the flag leaf.

### Impacts of drought on carbon metabolism of different photosynthetic organs

Drought has been known to have detrimental effects on leaf photosynthesis and metabolism (Wang et al., 2016a), with the extent of damage depending mainly, but not only, on the intensity of drought stress. For example, Zhao et al. (2019) reported that drought reduced subtending leaf photosynthesis, where photosynthetic rates were positively associated with soil water content for a given treatment. Zou et al. (2022a) observed that the mesophyll conductance limitations increased and significantly contributed to photosynthetic rate reductions of subtending leaf under short- or long-term drought stress conditions. Accordingly, in this study, moderate and severe drought decreased *Pn* of the subtending leaf in both cultivars, irrespective of their drought-tolerance with the decrease being significantly larger under severe compared to moderate drought stress conditions (**Figure 1**). Increasing drought stress severity resulted in a similar decreasing pattern in the subtending leaf’s chlorophyll content (**Supplementary Figure 3**), which was rather expected, taking in consideration the pivotal role of chlorophyll in the photosynthetic process (Eggink et al., 2001). In contrast to the subtending leaf, *Pn* and chlorophyll content of the capsule wall were markedly decreased only under conditions of severe drought stress (**Figure 1**; **Supplementary Figure 3**). This is in accordance with Gao et al. (2021) who also noted that drought stress has to be severe enough in order to result in reductions in chlorophyll content and *Pn* of the capsule wall since moderate drought had no effect on either trait in their study. Similar to the subtending leaf and the capsule wall, bract *Pn* were also decreased under severe drought stress conditions (**Figure 1**); however, contrastingly, those decreases were not accompanied by reductions in bract chlorophyll content (**Supplementary Figure 3**). Zhang et al. (2015) reported comparable results and speculated that this might be related to the restriction of CO_2_ assimilation in the bract. Evaluating stable carbon isotope abundance provides a way to evaluate source strength, and typically, lower *Pn* implies lower δ^13^C (Wu et al., 2019; Zhang et al., 2019). In our study, severe drought led to the reduction in δ^13^C values of all three photosynthetic organs in both cultivar of Dexiamian 1 and Yuzaomian 9110 compared to well-watered conditions (**Figure 2**), confirming the marked restrictions in CO_2_ assimilation that were indicated by the decreased *Pn*. Moreover, the reduction observed in the δ^13^C value, and consequently CO_2_ assimilation, of the subtending leaf decrease was much larger, compared to that of the bract and/or the capsule wall, and this could partially explain the decrease in the relative photosynthetic contribution of the subtending leaf to the boll weight under severe drought that was discovered in our study. Furthermore, larger decreases in *Pn* and δ^13^C values of subtending leaf were found in Yuzaomian 9110 than Dexiamian 1 under severe drought (**Figures 1**, **2**), indicating the photosynthetic assimilation of Yuzaomian 9110 was more sensitive to drought than that of Dexiamian 1.

Carbon assimilation is greatly dependent on Rubisco activity (Parry et al., 2013). In our study, Rubisco activity of the three photosynthetic organs was significantly decreased under severe drought conditions (**Figure 4**), confirming that CO_2_ fixation and formation of 3-phosphoglycerate was substantially constrained. Because sucrose is the primary photosynthate synthesized for subsequent transport to sink organs like the cotton boll (Zahoor et al., 2017a), one would anticipate a reduction in its content for source leaves exposed to photosynthesis-limiting drought. Nevertheless, despite the reduced CO_2_ fixation, sucrose contents of the subtending leaf, at 10 DPA and 31 DPA, as well as the bract and capsule wall at 31 DPA, were significantly increased (**Figure 3**). Accumulation of carbohydrates, in photosynthetic and non-photosynthetic organs under drought stress conditions, is a common mechanism of plants to maintain cell turgor and prevent cell dehydration (Liu et al., 2004; Muller et al., 2011; Durand et al., 2016). Especially in cotton, several studies have noted increases in sucrose under drought stress conditions and they have been credited with alterations in sucrose metabolizing enzymes activities (Zou et al., 2022b) or reductions in sucrose translocation (Lemoine et al., 2013). SuSy is most often considered to catalyse the reaction of sucrose decomposition in sink organs (Winter et al., 1997), however, SuSy activity of photosynthetic organs was measured in the synthetic sucrose direction of this study. In the current study, the activities of sucrose-synthesizing enzymes, SPS and SuSy, and sucrose-hydrolyzing enzymes, acid and alkaline invertase, were significantly increased in all three photosynthesizing tissues of drought stressed plants, compared to the control (**Figures 5**, **6**). In partial agreement with our results, Pilon (2015) found that carbohydrate metabolism in cotton leaves was compromised by water-deficit conditions. Zahoor et al. (2017a) observed substantial increases in SPS and SuSy activities of drought-stressed cotton plants, accompanied by concomitant increases in leaf sucrose content. As mentioned above however, sucrose translocation out of photosynthesizing tissues also determines tissue sucrose content (Koch, 2004). In our study, expression of *GhSUT1*, encoding a sucrose transporter in the subtending leaf, was down-regulated at all sampling dates under severe drought stress (**Figure 8**), indicating that a likely reduction in sucrose export and consequently resulting in sucrose accumulation in drought-stressed subtending leaf. Expression of *GhSUT1* in the bract and capsule wall was not affected at 10 days into the stress; however, it was substantially lower at 31 days into the stress compared to the control. At 10 DPA, sucrose content in the bract was not influenced by drought, but sucrose content in the capsule wall was significantly lower under severe drought (**Figure 3**), indicating that drought-induced changes in sucrose content of bracts and capsule walls during this period are governed by the balance between sucrose synthesis and degradation, not transport. However, sucrose contents of bract and capsule wall were increased at 31 DPA (**Figure 3**), indicating that despite the delayed response, compared to the subtending leaf, severe drought stress markedly inhibited sucrose export from the bract and capsule wall resulting in substantial increases in their sucrose content. Taking in consideration that sucrose translocation from the bract and capsule wall was only observed at 31 days into stress, a time point at which boll development has been almost completed, we speculate that this might be the reason why the photosynthetic contribution of the bract and capsule wall to boll weight was not decreased or even increased under severe drought stress.

In support of our speculation, after feeding the bract or the capsule wall at 10 DPA with ^13^CO_2_, the ratio of ^13^C allocated to other organs was not altered by severe drought (**Figure 7**). Contrastingly, after feeding the subtending leaf with ^13^CO_2_, the ratio of ^13^C content of corresponding bract plus boll (capsule wall, seed, fiber) to total ^13^C content (subtending leaf, bract plus boll) was markedly decreased under severe drought stress, compared to CK (**Figure 7A**), indicating that, among the three photosynthetic organs, only the export of photosynthate from the subtending leaf to other organs was restricted by severe drought at 10 and 31 DPA. In accordance with our results, Zahoor et al. (2017a) observed that water stress reduced leaf photo-assimilates partitioning towards other organs. Furthermore, only under severe drought was the distribution of ^13^C from the subtending leaf to seed and fiber decreased (**Figure 7B**), meaning that the photosynthate distribution within these non-leaf organs was not readily affected by drought stress. Bearing in mind that both the bracts and the capsule wall are closer to the seeds and fiber, compared to the subtending leaf, we speculate that the shorter distance the photosynthate produced by these non-leaf organs needs to cross (Hu et al., 2012) might be the reason why the relative photosynthetic contribution of the subtending leaf to boll weight was the only one to decrease under drought. This is in agreement with our previous conclusion that the subtending leaf is more sensitive to drought stress than non-leaf tissues.

## Conclusion

**Figure 10:**
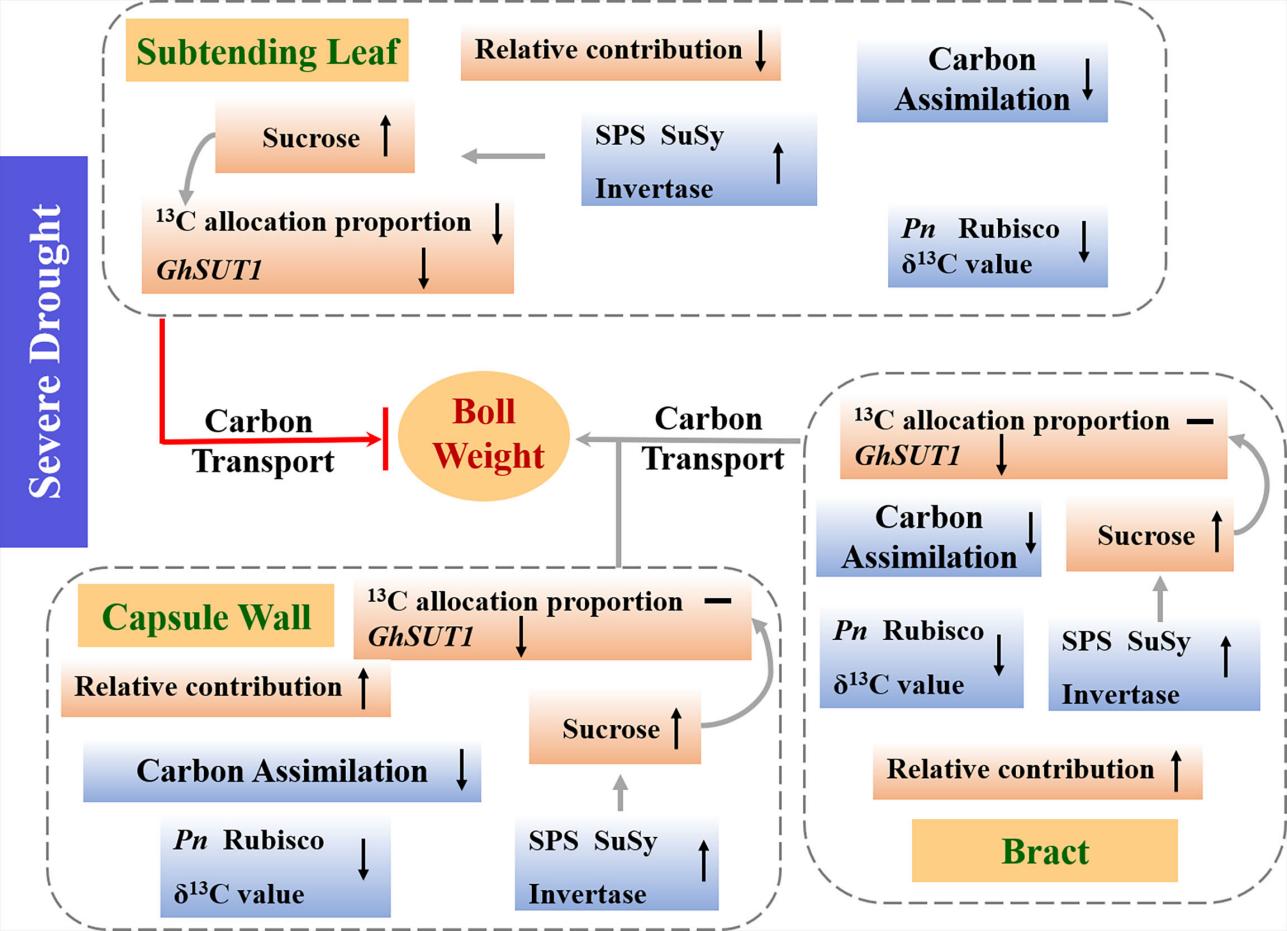
A schematic model depicting the mechanistic drivers of boll weight reductions under severe drought. Metabolism and parameters that increased, decreased or were unchanged under severe drought were indicated by “↑, “↓” or “—“, respectively. *Pn*: net photosynthetic rate, rubisco: ribulose-1,5-bisphosphate carboxylase, SPS: sucrose phosphate synthase, SuSy: sucrose synthase, *GhSUT1*: sucrose transporter gene.

The results, summarized in **Figure 10**, indicate that severe drought limited boll mass primarily due to reductions in carbon assimilation of the subtending leaf, bract and capsule wall and subsequent allocation to developing sink tissues. The decreases of *Pn* and δ^13^C value were observed in the subtending leaf, bract and capsule wall under severe drought. However, sucrose, content in leaves, bracts, and bolls were differentially affected under severe drought, due to the interactions between activities of SPS, SuSy, acid and alkaline invertases and changes in the expression of *GhSUT1*. Moreover, among the three photosynthetic organs, only distribution of photosynthate from the subtending leaf to seeds and fibers was restricted by drought. Taking in consideration that boll weight decreased, even though the contributions of the bracts and capsule wall were increased, we are led to suggest that the subtending leaf played the dominant role in boll weight loss under drought, due to decreases in carbon assimilation, perturbations in sucrose metabolism and inhibition of sucrose transport. Although Dexiamian 1 and Yuzaomian 9110 had different drought tolerance, both cultivars were explained by the same carbon assimilation and distribution pattern presented in **Figure 10**. In conclusion, drought-stable photosynthetic assimilation of the subtending leaf coupled with efficient sucrose synthesis and transport to developing sinks may be an important functional trait for improving drought tolerance.

## Data availability statement

The original contributions presented in the study are included in the article/**Supplementary Material**. Further inquiries can be directed to the corresponding author.

## Author contributions

ZZ, WH and YW conceived and designed the research. JZ, HZ, YL and JH conducted the experiments and collected the data. JZ analyzed the data and wrote the paper. WH, DL and JS commented and revised the paper. All authors contributed to the manuscript and approved the submitted version.

## Funding

This work was supported by the National Natural Science Foundation of China (31630051, 31901463), China Agriculture Research System of MOF and MARA (CARS-15-14), Natural Science Foundation of Jiangsu Province (BK20190524), the China Postdoctoral Science Foundation (2020M681633), Jiangsu Collaborative Innovation Center for Modern Crop Production (JCIC-MCP) and High-level Talent Introduction Program of Nanjing Agricultural University.

## Conflict of interest

The authors declare that the research was conducted in the absence of any commercial or financial relationships that could be construed as a potential conflict of interest.

## Publisher’s note

All claims expressed in this article are solely those of the authors and do not necessarily represent those of their affiliated organizations, or those of the publisher, the editors and the reviewers. Any product that may be evaluated in this article, or claim that may be made by its manufacturer, is not guaranteed or endorsed by the publisher.

## References

[B1] Abdelraheem A. Esmaeili N. O’connell M. Zhang J. (2019). Progress and perspective on drought and salt stress tolerance in cotton. Ind. Crops Prod. 130, 118–129. doi: 10.1016/j.indcrop.2018.12.070

[B2] Ai X. Liang Y. Wang J. Zheng J. Gong Z. Guo J. . (2017). Genetic diversity and structure of elite cotton germplasm (*Gossypium hirsutum* l.) using genome-wide SNP data. Genetica 145, 409–416. doi: 10.1007/s10709-017-9976-8 28755130

[B3] Araus J. L. Brown H. R. Febrero A. Bort J. Serret M. D. (1993). Ear photosynthesis, carbon isotope discrimination and the contribution of respiratory CO_2_ to differences in grain mass in durum wheat. Plant Cell Environ. 16, 383–392. doi: 10.1111/j.1365-3040.1993.tb00884.x

[B4] Bahrami F. Arzani A. Karimi V. (2014). Evaluation of yield-based drought tolerance indices for screening safflower genotypes. Agron. J. 106, 1219–1224. doi: 10.2134/agronj13.0387

[B5] Baker R. F. Leach K. A. Braun D. M. (2012). SWEET as sugar: New sucrose effluxers in plants. Mol. Plant 5, 766–768. doi: 10.1093/mp/sss054 22815540

[B6] Braun D. M. (2012). SWEET! the pathway is complete. Science 335, 173–174. doi: 10.1126/science.1216828 22246760

[B7] Chastain D. R. Snider J. L. Choinski J. S. Collins G. D. Perry C. D. Whitaker J. . (2016). Leaf ontogeny strongly influences photosynthetic tolerance to drought and high temperature in *Gossypium hirsutum* . J. Plant Physiol. 199, 18–28. doi: 10.1016/j.jplph.2016.05.003 27302003

[B8] Chastain D. R. Snider J. L. Collins G. D. Perry C. D. Whitaker J. Byrd S. A. (2014). Water deficit in field-grown *Gossypium hirsutum* primarily limits net photosynthesis by decreasing stomatal conductance, increasing photorespiration, and increasing the ratio of dark respiration to gross photosynthesis. J. Plant Physiol. 171, 1576–1585. doi: 10.1016/j.jplph.2014.07.014 25151126

[B9] Chen L. Cheung L. S. Feng L. Tanner W. Frommer W. B. (2015). Transport of sugars. Annu. Rev. Biochem. 84, 865–894. doi: 10.1146/annurev-biochem-060614-033904 25747398

[B10] Dağdelen N. Başal H. Yilmaz E. Gürbüz T. Akçay S. (2009). Different drip irrigation regimes affect cotton yield, water use efficiency and fiber quality in western Turkey. Agric. Water Manage. 96, 111–120. doi: 10.1016/j.agwat.2008.07.003

[B11] Dean C. Pichersky E. Dunsmuir P. (1989). Structure, evolution, and regulation of *rbcS* genes in higher plants. Annu. Rev. Plant Biol. 40, 415–439. doi: 10.1146/annurev.arplant.40.1.415

[B12] Ding X. Li X. Wang L. Zeng J. Huang L. Xiong L. . (2021). Sucrose-enhanced reactive oxygen species generation promotes cotton fiber initiation and secondary cell wall deposition. Plant Biotechnol. J. 19, 1092–1094. doi: 10.1111/pbi.13594 33787060 PMC8196644

[B13] Durand M. Porcheron B. Hennion N. Maurousset L. Lemoine R. Pourtau N. (2016). Water deficit enhances c export to the roots in *Arabidopsis thaliana* plants with contribution of sucrose transporters in both shoot and roots. Plant Physiol. 170, 1460–1479. doi: 10.1104/pp.15.01926 26802041 PMC4775148

[B14] Eggink L. L. Park H. Hoober J. K. (2001). The role of chlorophyll b in photosynthesis: Hypothesis. BMC Plant Biol. 1, 1–7. doi: 10.1186/1471-2229-1-2 11710960 PMC59834

[B15] Elmore C. D. (1973). Contributions of the capsule wall and bracts to the developing cotton fruit. Crop Sci. 13, 751–752. doi: 10.2135/cropsci1973.0011183X001300060049x

[B16] Gao H. Li N. Li J. Khan A. Ahmad I. Wang Y. . (2021). Improving boll capsule wall, subtending leaves anatomy and photosynthetic capacity can increase seed cotton yield under limited drip irrigation systems. Ind. Crops Prod. 161, 113–214. doi: 10.1016/j.indcrop.2020.113214

[B17] Gao M. Xu B. Wang Y. Zhou Z. Hu W. (2020). Quantifying individual and interactive effects of elevated temperature and drought stress on cotton yield and fibre quality. J. Agron. Crop Sci. 207, 422–436. doi: 10.1111/jac.12462

[B18] Hendrix D. L. (1993). Rapid extraction and analysis of nonstructural carbohydrates in plant tissues. Crop Sci. 33, 1306–1311. doi: 10.2135/cropsci1993.0011183X003300060037x

[B19] Hendrix D. L. Huber S. C. (1986). Diurnal fluctuations in cotton leaf carbon export, carbohydrate content, and sucrose synthesizing enzymes. Plant Physiol. 81, 584–586. doi: 10.1104/pp.81.2.584 16664860 PMC1075380

[B20] Hu Y. Oguchi R. Yamori W. Caemmerer S. V. Chow W. S. Zhang W. (2013). Cotton bracts are adapted to a microenvironment of concentrated CO_2_ produced by rapid fruit respiration. Ann. Bot. 112, 31–40. doi: 10.1093/aob/mct091 23625144 PMC3690982

[B21] Hu W. Snider J. L. Wang H. Zhou Z. Chastain D. R. Whitaker J. . (2018). Water-induced variation in yield and quality can be explained by altered yield component contributions in field-grown cotton. Field Crops Res. 224, 139–147. doi: 10.1016/j.fcr.2018.05.013

[B22] Hu W. Yang J. Meng Y. Wang Y. Chen B. Zhao W. . (2015). Potassium application affects carbohydrate metabolism in the leaf subtending the cotton (*Gossypium hirsutum* l.) boll and its relationship with boll biomass. Field Crops Res. 179, 120–131. doi: 10.1016/j.fcr.2015.04.017

[B23] Hu Y. Zhang Y. Li W. Oguchi R. Chow W. S. Luo H. . (2012). Important photosynthetic contribution from the non-foliar green organs in cotton at the late growth stage. Planta 235, 325–336. doi: 10.1007/s00425-011-1511-z 21904871

[B24] Hu Y. Zhang Y. Yi X. Zhan D. Luo H. Soon C. W. . (2014). The relative contribution of non-foliar organs of cotton to yield and related physiological characteristics under water deficit. J. Integr. Agric. 13, 975–989. doi: 10.1016/s2095-3119(13)60568-7

[B25] Kaur K. Gupta A. K. Kaur N. (2007). Effect of water deficit on carbohydrate status and enzymes of carbohydrate metabolism in seedlings of wheat cultivars. Indian J. Biochem. Biophys. 44, 223–230. doi: 10.1134/S1607672907040175 17970280

[B26] Klemens P. Patzke K. Deitmer J. Spinner L. Hir R. L. Bellini C. . (2013). Overexpression of the vacuolar sugar carrier *AtSWEET16* modifies germination, growth, and stress tolerance in arabidopsis. Plant Physiol. 163, 1338–1352. doi: 10.1104/pp.113.224972 24028846 PMC3813654

[B27] Koch K. (2004). Sucrose metabolism: regulatory mechanisms and pivotal roles in sugar sensing and plant development. Curr. Opin. Plant Biol. 7, 235–246. doi: 10.1016/j.pbi.2004.03.014 15134743

[B28] Lemoine R. Camera S. L. Atanassova R. Dédaldéchamp F. Allario T. Pourtau N. . (2013). Source-to-sink transport of sugar and regulation by environmental factors. Front. Plant Sci. 4. doi: 10.3389/fpls.2013.00272 PMC3721551 23898339

[B29] Liu F. Jensen C. R. Andersen M. N. (2004). Drought stress effect on carbohydrate concentration in soybean leaves and pods during early reproductive development: its implication in altering pod set. Field Crops Res. 86, 1–13. doi: 10.1016/s0378-4290(03)00165-5

[B30] Liu J. Ma Y. Lv F. Chen J. Zhou Z. Wang Y. . (2013). Changes of sucrose metabolism in leaf subtending to cotton boll under cool temperature due to late planting. Field Crops Res. 144, 200–211. doi: 10.1016/j.fcr.2013.02.003

[B31] Liu R. Zhou Z. Guo W. Chen B. Oosterhuis D. M. (2008). Effects of n fertilization on root development and activity of water-stressed cotton (*Gossypium hirsutum* l.) plants. Agric. Water Manage. 95, 1261–1270. doi: 10.1016/j.agwat.2008.05.002

[B32] Lokhande S. Reddy K. R. (2014). Reproductive and fiber quality responses of upland cotton to moisture deficiency. Agron. J. 106, 1060–1069. doi: 10.2134/agronj13.0537

[B33] Mehrabadi H. R. Nezami A. Kafi M. Ramezani Moghadam M. R. (2015). Yield, yield components, correlation coefficients and path analysis of cotton cultivars under drought stress. J. Crop Prod Proc. 5, 217–228. doi: 10.18869/acadpub.jcpp.5.17.217

[B34] Moran R. (1982). Formulae for determination of chlorophyllous pigments extracted with *N*,*N*-dimethylformamide. Plant Physiol. 69, 1376–1381. doi: 10.1104/pp.69.6.1376 16662407 PMC426422

[B35] Muller B. Pantin F. Génard M. Turc O. Freixes S. Piques M. . (2011). Water deficits uncouple growth from photosynthesis, increase c content, and modify the relationships between c and growth in sink organs. J. Exp. Bot. 62, 1715–1729. doi: 10.1093/jxb/erq438 21239376

[B36] Parida A. K. Dagaonkar V. S. Phalak M. S. Umalkar G. V. Aurangabadkar L. P. (2007). Alterations in photosynthetic pigments, protein and osmotic components in cotton genotypes subjected to short-term drought stress followed by recovery. Plant Biotechnol. Rep. 1, 37–48. doi: 10.1007/s11816-006-0004-1

[B37] Parry M. Andralojc P. J. Khan S. Lea P. J. Keys A. J. (2002). Rubisco activity: effects of drought stress. Ann. Bot. 89, 833–839. doi: 10.1093/aob/mcf103 12102509 PMC4233807

[B38] Parry M. A. Andralojc P. J. Scales J. C. Salvucci M. E. Carmo-Silva A. E. Alonso H. . (2013). Rubisco activity and regulation as targets for crop improvement. J. Exp. Bot. 64, 717–730. doi: 10.1093/jxb/ers336 23162118

[B39] Pilon C. (2015). Physiological responses of cotton genotypes to water-deficit stress during reproductive development (University of Arkansas: Graduate Dissertation).

[B40] Pugh D. A. Offler C. E. Talbot M. J. Ruan Y. (2010). Evidence for the role of transfer cells in the evolutionary increase in seed and fiber biomass yield in cotton. Mol. Plant 3, 1075–1086. doi: 10.1093/mp/ssq054 20864453

[B41] Riaz M. Farooq J. Sakhawat G. Mahmood A. Sadiq M. A. Yaseen M. (2013). Genotypic variability for root/shoot parameters under water stress in some advanced lines of cotton (*Gossypium hirsutum* l.). Gen. Mol. Res. 12, 552–561. doi: 10.4238/2013.February.27.4 23512672

[B42] Ruehr N. K. Offermann C. A. Gessler A. Winkler J. B. Ferrio J. P. Buchmann N. . (2009). Drought effects on allocation of recent carbon: from beech leaves to soil CO_2_ efflux. New Phytol. 184, 950–961. doi: 10.1111/j.1469-8137.2009.03044.x 19843305

[B43] Sauer N. (2007). Molecular physiology of higher plant sucrose transporters. FEBS Lett. 581, 2309–2317. doi: 10.1016/j.febslet.2007.03.048 17434165

[B44] Sharma B. Mills C. I. Snowden C. Ritchie G. L. (2015). Contribution of boll mass and boll number to irrigated cotton yield. Agron. J. 107, 1845–1853. doi: 10.2134/agronj15.0024

[B45] Shu H. Zhou Z. Xu N. Wang Y. Zheng M. (2009). Sucrose metabolism in cotton (*Gossypium hirsutum* l.) fibre under low temperature during fibre development. Eur. J. Agron. 31, 61–68. doi: 10.1016/j.eja.2009.03.004

[B46] Sicher R. Bunce J. Barnaby J. Bailey B. (2015). Water-deficiency effects on single leaf gas exchange and on C_4_ pathway enzymes of maize genotypes with differing abiotic stress tolerance. Photosynthetica 53, 3–10. doi: 10.1007/s11099-015-0074-9

[B47] Simkin A. J. Faralli M. Ramamoorthy S. Lawson T. (2019). Photosynthesis in non-foliar tissues: implications for yield. Plant J. 101, 1001–1015. doi: 10.1111/tpj.14633 PMC7064926 31802560

[B48] Wang H. Chen Y. Hu W. Wang S. Snider J. L. Zhou Z. (2017). Carbohydrate metabolism in the subtending leaf cross-acclimates to waterlogging and elevated temperature stress and influences boll biomass in cotton (*Gossypium hirsutum*). Physiol. Plant 161, 339–354. doi: 10.1111/ppl.12592 28581029

[B49] Wang Y. Chen M. Liang F. Tian J. Zhang Y. Jiang C. . (2021). Photosynthates competition within the boll-leaf system is alleviated with the improvement of photosynthetic performance during the succession of xinjiang cotton cultivars. Ind. Crops Prod. 160, 113–121. doi: 10.1016/j.indcrop.2020.113121

[B50] Wang R. Gao M. Ji S. Wang S. Meng Y. Zhou Z. (2016a). Carbon allocation, osmotic adjustment, antioxidant capacity and growth in cotton under long-term soil drought during flowering and boll-forming period. Plant Physiol. Biochem. 107, 137–146. doi: 10.1016/j.plaphy.2016.05.035 27288990

[B51] Wang R. Ji S. Zhang P. Meng Y. Wang Y. Chen B. . (2016b). Drought effects on cotton yield and fiber quality on different fruiting branches. Crop Sci. 56, 1265–1276. doi: 10.2135/cropsci2015.08.0477

[B52] Wang Y. Zhang Y. Han J. Li C. Wang R. Zhang Y. . (2019). Improve plant photosynthesis by a new slow-release carbon dioxide gas fertilizer. ACS Omega 4, 10354–10361. doi: 10.1021/acsomega.8b03086 31460129 PMC6648988

[B53] Winter H. Huber J. L. Huber S. C. (1997). Membrane association of sucrose synthase: changes during the graviresponse and possible control by protein phosphorylation. FEBS Lett. 420, 151–155. doi: 10.1016/S0014-5793(97)01506-8 9459300

[B54] Wu A. Hammer G. L. Doherty A. Caemmerer S. V. Farquhar G. D. (2019). Quantifying impacts of enhancing photosynthesis on crop yield. Nat. Plants 5, 380–388. doi: 10.1038/s41477-019-0398-8 30962528

[B55] Wullschleger S. D. Oosterhuis D. M. (1990). Photosynthetic carbon production and use by developing cotton leaves and bolls. Crop Sci. 30, 1259–1264. doi: 10.2135/cropsci1990.0011183X003000060021x

[B56] Zahoor R. Dong H. Abid M. Zhao W. Wang Y. Zhou Z. (2017a). Potassium fertilizer improves drought stress alleviation potential in cotton by enhancing photosynthesis and carbohydrate metabolism. Environ. Exp. Bot. 137, 73–83. doi: 10.1016/j.envexpbot.2017.02.002

[B57] Zahoor R. Zhao W. Dong H. Snider J. L. Abid M. Iqbal B. . (2017b). Potassium improves photosynthetic tolerance to and recovery from episodic drought stress in functional leaves of cotton (*Gossypium hirsutum* l.). Plant Physiol. Biochem. 119, 21–32. doi: 10.1016/j.plaphy.2017.08.011 28843133

[B58] Zhang H. Hartmann H. Gleixner G. Thoma M. Schwab V. F. (2019). Carbon isotope fractionation including photosynthetic and post-photosynthetic processes in C_3_ plants: Low [CO_2_] matters. Geochim. Cosmochim. Acta 245, 1–15. doi: 10.1016/j.gca.2018.09.035

[B59] Zhang Y. Zhang Y. Wang Z. Wang Z. (2011). Characteristics of canopy structure and contributions of non-leaf organs to yield in winter wheat under different irrigated conditions. Field Crops Res. 123, 187–195. doi: 10.1016/j.fcr.2011.04.014

[B60] Zhang C. Zhan D. Luo H. Zhang Y. Zhang W. (2015). Photorespiration and photoinhibition in the bracts of cotton under water stress. Photosynthetica 54, 12–18. doi: 10.1007/s11099-015-0139-9

[B61] Zhan D. Yang Y. Hu Y. Zhang Y. Luo H. Zhang W. (2014). Contributions of nonleaf organs to the yield of cotton grown with different water supply. Sci. World J. 2014, 602747. doi: 10.1155/2014/602747 PMC4058802 24982968

[B62] Zhao W. Wang R. Hu W. Zhou Z. (2019). Spatial difference of drought effect on photosynthesis of leaf subtending to cotton boll and its relationship with boll biomass. J. Agron. Crop Sci. 205, 263–273. doi: 10.1111/jac.12320

[B63] Zou J. Hu W. Li Y. He J. Zhu H. Zhou Z. (2020). Screening of drought resistance indices and evaluation of drought resistance in cotton (*Gossypium hirsutum* l.). J. Integr. Agric. 19, 495–508. doi: 10.1016/s2095-3119(19)62696-1

[B64] Zou J. Hu W. Li Y. Zhu H. He J. Wang Y. . (2022a). Leaf anatomical alterations reduce cotton’s mesophyll conductance under dynamic drought stress conditions. Plant J. 111, 391–405. doi: 10.1111/tpj.15794 35506315

[B65] Zou J. Snider J. L. Zhu H. He J. Li Y. Zhou Z. . (2022b). Soil drought duration and severity affect cotton boll biomass by altering recovery times and carbon dynamics of subtending leaf. J. Agron. Crop Sci. 00, 1–22. doi: 10.1111/jac.12595

