# Supplementary material for: Carbon assimilation and distribution in cotton photosynthetic organs is a limiting factor affecting boll weight formation under drought
Source: Front Plant Sci. 2022 Sep 21;13:1001940. doi: 10.3389/fpls.2022.1001940 (PMC9532866; doi:10.3389/fpls.2022.1001940)
Supplement: Supplementary file 1 [file DataSheet_1.docx]

Supplementary Material

# Supplementary Figures and Tables

## Supplementary Table

**Supplementary Table 1** Primer sequences used for qRT-PCR expression analysis.

| **Primer name** | **Gene description** | **Primer sequences** |
| --- | --- | --- |
| *SUT1* | Sucrose transport protein | Forward: TACTGACTGGATGGGCAAAG |
|  |  | Reverse: AACAAATAGCCAGCAGAAGG |
| *18S* | Housekeeping gene | Forward: TGACGGAGAATTAGGGTTCGA |
|  |  | Reverse: CCGTGTCAGGATTGGGTAATTT |

## Supplementary Figure


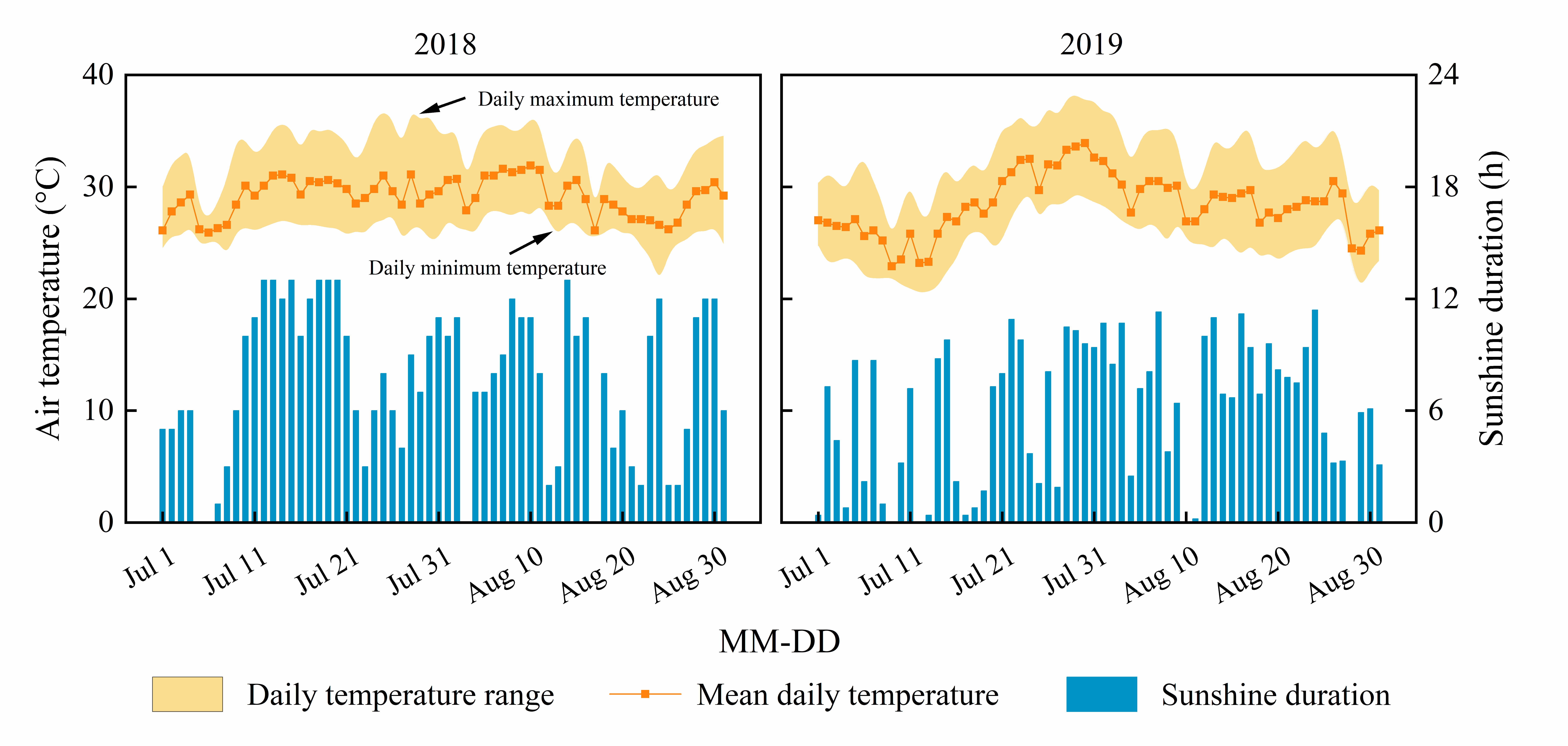


**Supplementary Figure 1.** Changes of daily maximum temperature, daily minimum temperature, mean daily temperature and sunshine duration during the cotton flowering and boll-forming period (July and August) in 2018 and 2019. MM-DD: month-day. All observations were provided by Nanjing Weather Station.





**Supplementary Figure 2.** Dynamics of soil relative water content (SRWC) in 2018 and 2019. Vertical bars denote standard error (n = 3).


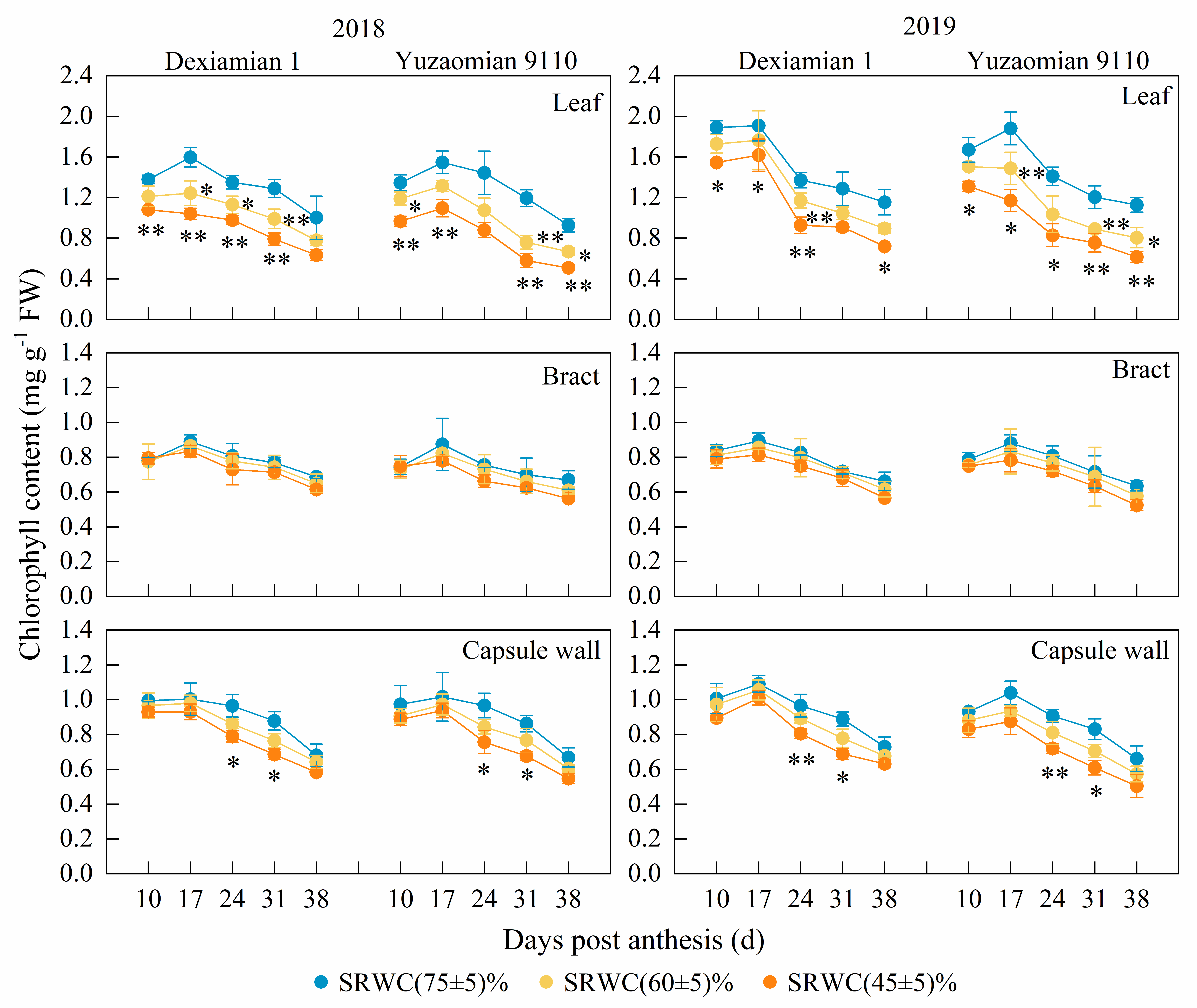


**Supplementary Figure 3.** Effects of drought on the chlorophyll content of the subtending leaf, bract and capsule wall in 2018 and 2019. Vertical bars denote standard error (n = 3). The asterisks indicate significantly differences between SRWC(75±5)% and SRWC(60±5)% or SRWC(45±5)% within each cultivar for a t-test (* *P* < 0.05, ** *P* < 0.01).
